# “Beyond the Bladder: Exploring the Intricacies of Emphysematous Cystitis and Its Surprising Associations”

**DOI:** 10.1155/2023/5451554

**Published:** 2023-08-08

**Authors:** Nishant Allena, Nismat Javed, SriKaran Bojja, Arundhati Dileep, Maryam Soliman

**Affiliations:** ^1^Division of Pulmonary Medicine and Critical Care, BronxCare Health System, New York, USA; ^2^Department of Internal Medicine, BronxCare Health System, New York, USA

## Abstract

Emphysematous cystitis is defined by the presence of air within the bladder wall or lumen in imaging studies with increased incidence in elderly women and diabetics. It is a result of gas-forming organisms like *Klebsiella* and *E. coli* but can be caused by fungi such as *Candida* and *Aspergillus* as well with a wide spectrum of clinical presentations. In this article, we present a case of a 77-year-old female with diabetes mellitus who presented to the hospital with a chief complaint of left lower quadrant abdominal pain. Abdominal imaging revealed emphysematous cystitis, paraspinal abscess, and air in the thoracic and lumbar vertebrae. Laboratory results showed leukocytosis, lactic acidosis, and urinalysis significant for urinary tract infection but no positive urine or blood cultures. The patient was admitted to the intensive care unit for septic shock and was treated with mechanical ventilation, vasopressor support, and prompt antimicrobial therapy following which the requirement of vasopressors ceased on the third day of admission. The decision was then made by the family to pursue hospice care, following which mechanical ventilation was discontinued and the patient transferred to inpatient hospice. With this case report, we aim to add to the existing literature regarding the spread of intra-abdominal infections and go over a brief review of the currently available literature. From our review, we would like to conclude that the presence of pneumoracchis, especially in the setting of an intra-abdominal infection, is a poor prognostic marker, and timely diagnosis and treatment of potential causes are required to reduce mortality.

## 1. Introduction

Emphysematous cystitis is defined by the presence of air within the bladder wall or lumen in imaging studies, with diabetes mellitus and elderly age being considered as major risk factors [1]. It is caused by gas-forming organisms, most commonly *Escherichia coli* and *Klebsiella pneumoniae*. Clinical presentation appears to be broad, ranging from asymptomatic to septic shock [1]. Pneumoracchis is a rare complication characterized by the presence of intraspinal air in imaging and is caused by traumatic, iatrogenic, and infectious etiologies [2]. Concomitant emphysematous cystitis and pneumoracchis are rare phenomena and have been associated with poor prognosis and reported mortality in the existing literature.

## 2. Case Presentation

A 77-year-old female with a medical history notable for hypertension, diabetes mellitus, and sciatica presented to the emergency department with lower left quadrant abdominal pain of 1 week duration, which has been gradually worsening with associated nausea, loss of appetite, and 1 episode of nonbilious/nonbloody vomiting before coming to the hospital. She also endorsed weakness in the legs leading to difficulty ambulating. Four days prior to this presentation, she presented to the emergency department with acute on chronic low back pain. She had left low back pain radiating to the left hip which felt like prior sciatica. At that time, she was treated with analgesics and was discharged on the same day. The patient's medical history was notable for hypertension, diabetes mellitus, and sciatica.

She was seen previously in the primary care clinic endorsing lower abdominal pain twice in the past year for similar reasons. Urine culture at that time grew *Klebsiella* *pneumoniae* > 100,000 CFU/ml which was pansensitive and treated with a course of oral cefuroxime.

On initial exam, the patient was in distress due to abdominal pain, and Spo2 was noted to be 84% following which the patient was put on a non-rebreather mask and admitted to the intensive care unit (ICU). On admission to the ICU, the patient was found to be in Atrial fibrillation with a rapid ventricular rate (RVR) with rate of 149 beats/min. The patient's blood pressure started to drop, central venous access was obtained, and vasopressors were administered.

Initial laboratory investigations revealed leukocytosis of 12 × 103 L with 89% neutrophil predominance and platelet count of 7 × 109/L. Metabolic panel revealed elevated BUN (63 mg/dL) and creatinine (1.6 mg/dL). Lactate of 5.1 mmol/L elevated LDH (409 U/L), C reactive protein (CRP) of 268.6 mg/L, ferritin (919 ng/ml), and haptoglobin (355 mg/dL). D-dimer was elevated to 1560 ng/ml. Elevated alkaline phosphatase (ALP) of (227 U/L) is mentioned in Table 1.

Urinalysis revealed turbid urine with 2+ proteinuria, heavy glycosuria, blood, bacteria with positive leukocyte esterase, and a moderate number of WBCs. Due to a high suspicion of septic shock secondary to a urinary tract infection (UTI), the patient was empirically started on vancomycin and piperacillin-tazobactam for broad-spectrum coverage.

**Figure 1:**
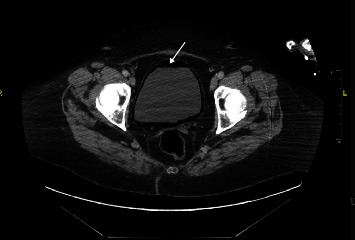
CT abdomen and pelvis showing emphysematous cystitis.

**Figure 2:**
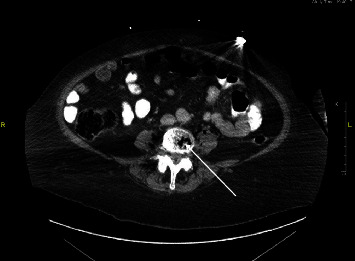
CT abdomen and pelvis showing pneumoracchis.

**Figure 3:**
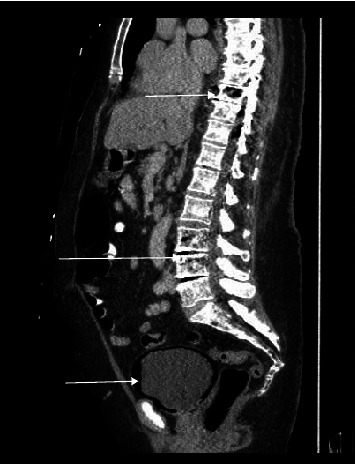
Saggital section on CT showing emphysematous cystitis and pneumoracchis.

CT (computed tomography) of the chest, abdomen, and pelvis as shown in Figures 1–3 were done revealing the following findings. Multiple lucencies most likely represent air in the T7, T8, T9, and T10 vertebrae associated with prevertebral and paravertebral abscesses along with air in the thoracic spinal canal. Multiple collections of air in the L3-L4 vertebral bodies associated with air in the prevertebral and paravertebral soft tissues are suggestive of a paraspinal abscess in the lumbar vertebral region. Multiple small collections of air are also seen in the left psoas muscle. There is air in the lumbar spinal canal surrounding the thecal sac with these findings concerning osteomyelitis involving the thoracic and lumbar vertebrae with paravertebral soft tissue components and intraspinal extension of the infection. Goitrous right thyroid lobe that is displacing the trachea to the left with mild compression of the tracheal lumen. The right thyroid lobe mass or goiter measures 5.74 × 5.69 × 4.78 cm. This shows heterogeneous enhancement. There was also evidence of extensive emphysematous cystitis.

Antibiotic coverage was targeted to cover for *Staphylococcus*, *Klebsiella*, *Proteus*, *Enterococcus*, *Pseudomonas aeruginosa*, and *Enterobacter* spp., source control could not be pursued due to severe thrombocytopenia and multilevel abscesses, and thus, the patient was not deemed a candidate for any surgical or Interventional radiology intervention. Therefore, the empiric antibiotic coverage was broadened to vancomycin with a target level of 15-20 ug/mL and meropenem along with metronidazole for additional anaerobic coverage. Severe thrombocytopenia was noted and she received an SD platelet transfusion with a poor increment in platelet count most likely secondary to a consumptive process. Disseminated intravascular coagulation (DIC) was ruled out and the recommendation by hematology was to transfuse platelets prophylactically until count recovery and to control the underlying disease process. The patient was started on antibiotics, and blood and urine cultures were sent. The admission was further complicated by new onset tonic-clonic seizures managed with benzodiazepines and levetiracetam.

However, despite all appropriate therapy, the patients' clinical status is concerned to worsen with the increasing requirement of vasopressors. A meeting was then conducted with the family who then agreed to pursue hospice care.

She was then taken off mechanical ventilation and transferred to an in-patient hospice unit.

## 3. Discussion

Emphysematous cystitis, a relatively rare condition primarily caused by gas-forming anaerobic organisms, has been documented globally with a number of cases ranging from 100 to 300 from 2007 to 2016 [1, 3]. Many cases with emphysematous cystitis have been reported as presented in Table 2.

### 3.1. Organisms and Risk Factors

The common pathogens involved include *Enterococcus coli* (40.4%), *Klebsiella pneumoniae* (34.6%), *Proteus mirabilis*, *Enterococcus*, *Pseudomonas aeruginosa*, and ESBL (36.5%) [13]. Risk factors for the development of emphysematous cystitis include age greater than 60 years, female sex, type 2 diabetes mellitus, malignancy, and neurogenic bladder [1, 14]. The increase in the number of comorbidities is also an essential factor towards the complicated course of the disease [6]. Additionally, recurrent urinary tract infections also increase the likelihood of surgical interventions [9]. Although our patient did not have any history of malignancy or neurogenic bladder, older age, female sex, and type 2 diabetes mellitus increased the likelihood of developing emphysematous cystitis.

### 3.2. Presentation

The spectrum of symptoms at presentation can range from patients being asymptomatic to a complicated course of illness, for example, sepsis and anuria [15–17]. Although abdominal pain and dysuria were relatively common presenting symptoms, altered mental status was observed in older individuals [5, 6, 10, 11]. Rapid deterioration of patients was relatively uncommon unless the presenting symptoms and signs were suggestive of impending shock [6]. In our case, the first symptom was in fact left abdominal pain followed by hemodynamic instability and acute hypoxic respiratory failure prior to the development of sepsis. Additionally, the development of arrhythmia in our case because of this sequence of events has also not been documented in prior studies. Furthermore, no cases of thrombocytopenia have been documented in patients presenting with emphysematous cystitis.

### 3.3. Imaging and Investigations

High degree of clinical suspicion is required to detect emphysematous cystitis. Although nonspecific findings can be seen on lab investigations including hematuria and pyuria, radiography continues to play an influential role in diagnosis [4–6]. Plain X-rays might provide a vague visualization of the characteristic findings, but CT scan is considered the investigation of choice for the disease because of better characterization of the severity and extent of the disease [18]. CT scan findings often show the presence of air, bladder wall thickening, or mottling of air bubbles in the wall in most case presentations [4, 5, 9–11]. Fumeo et al. reported the sensitivity and specificity of CT scan for emphysematous cystitis to be approximately 100% [18]. Cystoscopy evaluation is warranted in patients who have vague CT scan findings [19].

### 3.4. Treatment

The treatment strategy in such patients includes source control by reducing bacterial burden through appropriate antibiotic coverage and rapid disease debulking by surgical intervention [9, 20]. The antibiotic choices depend on the sensitivity analysis of the underlying organism and might range from ceftriaxone, piperacillin-tazobactam, and meropenem [4, 6–11]. In a few cases with complications, vancomycin, colistin, ertapenem, and gentamicin were also used [4, 8, 10]. Although antibiotic coverage was adequate in our patient, given the initial presentation of sepsis, the response to management was suboptimal and required surgical intervention. However, given the comorbidities and overall status of the patient, surgery was deemed high risk.

### 3.5. Complications

Complications of the disease can include bladder rupture, bladder dysfunction, obstruction of the urinary tract, which can eventually lead to bilateral renal dysfunction, and acute kidney injury that might require chronic hemodialysis and sepsis [19]. Emphysematous is also a rare complication that has been reported in 2 cases as a result of *Escherichia coli* and 4 cases as a result of *Klebsiella pneumoniae* [21–26]. The possible mechanism involves bacterial infiltration leading to bladder distension and microscopic tears transiently, allowing bacterial seeding and entry into the intramural space. Although the initial tears might heal, these bacteria undergo anaerobic respiration producing gaseous bubbles resulting in disruption of the serosa [27]. This process facilitates retroperitoneal spread, and the spread of the disease is faster in a patient with the focus of infection in the retroperitoneal organ, as in our case, the kidney. However, interestingly, our patient did not have positive urine or blood cultures for either *Klebsiella pneumoniae* or *Escherichia coli*.

Pneumoracchis is another rare complication of the disease. Usually, traumatic or iatrogenic causes are at play, but in a few cases, infection can also produce the same clinical picture. Bacteremia from an underlying organism can result in bacterial translocation and, subsequently, produce the spinal pathology [22]. Risk factors include old age and uncontrolled diabetes. Management of pneumoracchis depends upon treating the underlying cause; however, surgical intervention might be needed in case of spinal cord compression [21].

### 3.6. Prognosis and Mortality

Prognosis and mortality both depend upon the clinical spectrum of the condition. In patients with favourable presentations such as the absence of shock on presentation, absence of severe hypoalbuminemia, appropriate response to antibiotics, or lack of thrombocytopenia, the prognosis is generally better [28]. However, consistent with our case, patients presenting with shock on admission, altered mental status, and thrombocytopenia had a poor prognosis with the mortality rate as high as 52.4% [28].

## 4. Conclusion

Emphysematous cystitis is a rare but devastating disease with far-reaching consequences and complications if diagnosis or treatment is delayed. The spectrum of this disease is very vast, and more studies are needed to establish guidelines and criteria for timely diagnosis and optimal management so as to reduce complications.

## Figures and Tables

**Table 1:** Laboratory results on admission and two days after admission.

Variable	Ref. range	On presentation	48 hr after the presentation
WBC (×109/L)	4.8-10.8	12.0	17.3
RBC (×1012/L	4.00-5.20	4.98	4.15
HGB (g/L)	12.0-16.0	12.5	11.2
HCT (L/L)	42.0-51.0	42.3	35
PLT (×109/L)	150-400	7	13
Abs. neutrophils (×109/L)	1.5-8.0	89.0	90.3
pH venous	7.350-7.450	7.387	7.320
pCO2 venous (mmHg)	35.0-45.0	43.7	41.0
Bicarbonate venous (mmol/L)	22.0-28.0/24-30	25.7/	20.5/
Lactate (mmol/L)	0.5-1.6	5.1	1.2
D-dimer (ng/mL)	0-230	1560	2602

**Table 2:** Brief summary of recent cases with emphysematous cystitis.

Author	Presentation and comorbidities	Organism	Imaging and investigations	Treatment	Outcome
Adeyemi and Flaherty [4]	76 yo M with foul-smelling urine/T2DM	*Escherichia coli* and *Klebsiella pneumoniae*.	Urine culture positive, blood culture negative, CT scan-bladder wall thickening with air in the bladder wall. No pneumoracchis	IV piperacillin/tazobactam followed by ertapenem	Discharged after 14 days

Liao et al. [5]	65 yo F with dysuria and abdominal pain/HTN, T2DM	*Klebsiella pneumoniae*	Leukocytosis, hyperglycemia, lactic acidemia, acute kidney injury, urine cultures positive, and blood cultures negative. CT-bladder wall contoured by mottled air bubbles. No pneumoracchis	Bladder drainage and IV antibiotics	Discharged after 10 days

Zhang et al. [6]	66 yo M with confusion and jaundice/HTN, T2DM, PUD	*Klebsiella pneumoniae*	Leukocytosis, transaminitis, acute kidney injury, and pelvic CT-intramural gas formation in the bladder. No pneumoracchis	Complicated by septic shock and was being treated with meropenem	Died after massive resuscitation efforts

Suzuki et al. [7]	75 yo M/dementia	*Klebsiella pneumoniae*	Urine and blood cultures positive, leukocytosis, acute kidney injury, pyuria, CT-emphysematous prostatic abscess, emphysematous cystitis, and renal abscess. No pneumoracchis	Meropenem IV	Discharged

Nolazco et al. [8]	39 yo F/T2DM	*Klebsiella pneumoniae*	CT-gas in the bladder wall. No pneumoracchis	Vancomycin, meropenem, and colistin, bladder drainage, strict glycemic control	Discharged

Wang et al. [9]	62 yo M with right leg pain/T1DM, recurrent UTI	*Klebsiella pneumoniae*	CT-extensive subcutaneous air involving the entire right lower limb and tracking up into the pelvis, as well as air within the wall of the bladder and the prostate. Pneumoracchis present	Surgical intervention for fasciitis and repeat CT scan still showed air bubbles in the pelvis, was given IV piperacillin-tazobactam and underwent cystoprostatectomy with ileal conduit	Discharged after 13 days

Ahmed et al. [10]	95 yo M with altered mental status/T2DM	*Klebsiella pneumoniae*, *Streptococcus* pyogenes	CT-hydronephrosis, a thick-walled urinary bladder with diverticula and air in the bladder wall suggestive of emphysematous cystitis. No pneumoracchis	IV ceftriaxone and gentamicin	Discharged after 4 weeks

Bos et al. [11]	84 yo F with lower abdominal pain/T2DM	*Klebsiella pneumoniae* and *Enterococcus* species	CT-distended bladder measuring 10 × 9 cm with extensive peripheral air in its inner margin and a significant amount of gas anteriorly. No pneumoracchis	IV ceftriaxone	Discharged after 10 days

Wadekar et al. [12]	82 yo F with abdominal pain and vomiting/RA	*Escherichia coli*	CT-thickened urinary bladder with intramural air density. No pneumoracchis	Nitrofurantoin and amikacin	Discharged after 7 days

Our case report	77 yo F with abdominal pain and nausea/HTN, T2DM	*Klebsiella pneumoniae*	CT-multiple air densities and extensive emphysematous cystitis. Pneumoracchis present	Vancomycin, meropenem, and metronidazole	Transferred to in-patient hospice

## Data Availability

The data used to support the findings of this study are included within the article.
